# Auricular acupuncture as stress-relieving intervention for parents of infants in the neonatal intensive care unit: insights gained from a pilot study

**DOI:** 10.3389/fgwh.2025.1597105

**Published:** 2025-10-08

**Authors:** Helle Haslund-Thomsen, Bettina Svelle, Christina Skoda, Malene Horskjær, Marie Germund Nielsen

**Affiliations:** 1 Department of Clinical Medicine, Aalborg University, Aalborg, Denmark; 2 The Clinical Nursing Research Unit, Aalborg University Hospital, Aalborg, Denmark; 3 Department of Pediatrics and Adolescent Medicine, Aalborg University Hospital, Aalborg, Denmark; 4 Public Health and Epidemiology Group, Department of Health Science and Technology, Aalborg University, Aalborg, Denmark

**Keywords:** NADA auricular acupuncture, NICU, stress, parents, sleep, physical discomfort

## Abstract

**Background and aim:**

This pilot study aimed to explore the feasibility and effects of auricular acupuncture called NADA, according to the principles of the National Acupuncture Detoxification Association. Previous studies have reported the effects of NADA on stress, anxiety, and sleep. Given the high levels of stress, anxiety, and psychological distress commonly experienced by parents of infants admitted to the Neonatal Intensive Care Unit (NICU), the study sought to determine whether NADA could serve as a stress-relieving intervention.

**Method:**

The pilot study was conducted as an observational cross-sectional study for 6 months from October 2019. The “Nada in NICU” pilot project involved 41 parents (33 women and 8 men) who received between 3 and 16 NADA interventions during their child's NICU hospitalization. Data were collected through a questionnaire assessing sleep, stress/restlessness, and physical well-being. Participants were also given the option to add free-text comments in the questionnaire regarding their experiences with the NADA treatment. Quantitative data were analyzed using mixed regression, while qualitative data were thematically analyzed to identify recurring themes.

**Findings:**

The results indicated a statistically significant reduction in stress, sleep disturbances, and physical discomfort post-NADA intervention, with a mean difference in sleep scores of −1.951. Qualitative feedback generated an overall theme, “An increased feeling of calmness,” and two themes, “A psychological booster,” reflecting parents’ experiences of enhanced mental clarity and emotional regulation and “Bodily calmness,” highlighting improved physical relaxation and sleep quality. Parents universally regarded NADA as a relevant and beneficial intervention during their NICU stay.

## 1 Background

When an infant is born preterm and requires hospitalization in a neonatal intensive care unit (NICU), parents often experience an array of negative emotions, including stress and anxiety, which are related to the infant's condition, parenting roles, and the NICU environment and staff (1). A recent meta-analysis showed that 49% of mothers and 23% of fathers experienced anxiety following their infant’s admission to the NICU (2). Most hospitalized parents are affected to varying degrees of anxiety and stress during their child's admission to the NICU, while still being expected to undertake their parental responsibilities. In a study by Diaz-Caneja et al. (3), parents described their experience as similar to a grief reaction, including shock or disbelief, guilt and blame, and avoidance and escape strategies. These parents are at increased risk of developing depression, which can negatively impact their well-being during hospitalization and after discharge (4). Furthermore, parents suffer negative psychological effects during and after NICU, including disrupted parent–infant attachment (5) and an affected sense of parental role which have been associated with symptoms of depression negatively affecting parental well-being (4). Additionally, patients of hospitalized infants report higher rates of anxiety and posttraumatic stress compared with parents of healthy infants (6–8).

It is also known that these psychological constraints negatively affect milk production, potentially compromising mothers' ability to feed their infants in accordance with recommendations on human milk nutrition (9). A study by Ziomkiewicz et al. (10) found that perinatal psychosocial stress negatively affected the energy density and fatty acid content in breast milk.

Furthermore, infants in the NICU are at a heightened risk for adverse developmental, cognitive, academic, and mental health outcomes due to prematurity, illness, quality of care, and mother–infant interaction (11). Parent–infant closeness evolves and is influenced by multifaceted biopsychosocial factors (12). Accordingly, parental stress affecting mother–infant interaction and quality may negatively impact these infants both during NICU hospitalization and after discharge (8).

Supporting parents in the NICU after preterm birth is crucial not only for their own mental health but also due to potential negative implications for the parent–infant relationship and the child’s subsequent development. A multilayered approach to supporting parents of preterm infants in the NICU is therefore recommended. Evidence specifically supports the inclusion of individual psychological and psychosocial support, peer-to-peer support, and family-centered care (7).

### 1.1 NADA acupuncture

To relieve stress and support recovery, auricular acupuncture, which originated from traditional Chinese medicine, was adapted and further developed in the USA in the 1970s. American psychiatrist Michael Smith developed an auricular acupuncture model, later known as the NADA (National Acupuncture Detoxification Association) method (13). A NADA intervention session involves the placement of a total of 10 small needles, five in each ear, at specific points. The needles are then left *in situ* for 45 min while the individual is in a calm environment.

NADA has been used in various contexts and populations. Notably, it has been applied in substance abuse treatment programs, hospitals, and prisons for almost 40 years despite conflicting and limited evidence regarding its effectiveness (14). NADA has been used to treat posttraumatic stress disorder (PTSD), for example, in refugee settings (15) and has been found to reduce burnout and distress. A study by Olshan-Perlmutter et al. (16) found that auricular acupuncture reduced symptoms of anxiety and burnout in behavioral healthcare providers. NADA has also been used to treat maternal perinatal anxiety. In the study by Favre-Fèlix et al. (17), NADA was found to reduce maternal anxiety levels upon arrival in the operating room and immediately before the commencement of a cesarean section, compared with usual care.

A meta-analysis indicated that NADA is effective in treating insomnia. However, due to the low quality of existing trials, further clinical trials with higher design quality, extended treatment duration, and longer follow-up periods are necessary (18). To date, no studies on the effects and experiences with NADA as a stress-relieving intervention for parents of infants admitted to the NICU have been reported.

Given its reported effects on psychological stress and sleep (19), we anticipated that NADA could be valuable to support parents in handling the stressful, sleep-disturbed, and often traumatic time during their NICU stay. In this study, we aimed to pilot test NADA to generate knowledge about the feasibility of the intervention and its effects and parents' experiences with it in a NICU setting.

## 2 Method

In October 2019, the “NADA in NICU” pilot project was started. Two experienced neonatal nurses were certified as NADA therapists at the association called NADA Denmark. NADA was offered as an individual intervention at flexible times. We aimed to test NADA in a form that was feasible in everyday clinical life and adaptable to the rhythm, physical condition, and general needs of both the parents and infants, with a focus on creating a peaceful moment for the parents to receive the intervention. Posters with information about the NADA intervention were strategically placed in prominent locations within the NICU to inform parents of its availability during their infant's hospitalization. In addition to this passive recruitment strategy, nursing staff actively engaged with parents by verbally informing them about the pilot testing of the NADA intervention. The two NADA therapists also personally informed parents that NADA was offered only at times when they were present in the NICU and had allocated time for NADA sessions. Through this combined approach, parents were recruited via visual materials and direct communication. There were no formal exclusion criteria for participation. Instead, parents independently assessed their own readiness and interest in receiving the NADA intervention based on their individual circumstances during their infant's hospitalization in the NICU. Neither the length of the NICU stay nor the timing of the intervention served as limiting factors, as the aim was to explore how NADA was experienced across a diverse parental population.

This pilot study was conducted as an observational cross-sectional study. Data were collected using a questionnaire developed to investigate the experience of NADA based on three general themes: sleep, stress/restlessness, and physical well-being (see Appendix 1). These three themes cover common concerns experienced by parents of children admitted to the NICU (3). For each of the three themes, one question was developed. The response categories followed a Likert scale format but lacked descriptive labels, e.g., “strongly agree” and “disagree.” Furthermore, the parents were asked whether they considered the offer of NADA during hospitalization to be relevant in the NICU.

Parents were allowed to provide written comments regarding their experiences with receiving NADA as part of completing the evaluation questionnaire. These written comments were chosen to add new perspectives as a supplement to the scorings on the questionnaire evaluating the NADA treatment and provided an opportunity for parents to express their experiences in their own words.

The NADA therapists filled in information about the physical setting during the NADA intervention and how it was experienced by the parent, whether the session was best conducted in a separate room or in the patient room, in a bed or a chair, and without or with the child or others in the room. This was conducted to gather knowledge about their experiences with NADA in different physical contexts. This is important knowledge for potential further implementation of the intervention in the NICU. Various calm settings such as available empty rooms and rooms used for appointments and conversations with a social worker, a psychologist, or a priest, were used pragmatically depending on what was possible in the ward at a time that was convenient for parents to receive NADA. These rooms were equipped with a comfortable reclining chair or a bed.

In the context of providing NADA to parents of children admitted to the NICU, several ethical considerations had to be addressed. NADA has demonstrated efficacy in reducing stress and anxiety levels, making it a pertinent intervention to investigate within this specific demographic context. The decision to offer NADA to parents was grounded in the understanding that they were experiencing significant emotional challenges during their child's hospitalization, and thus, exploring potential supportive interventions was important.

Written informed consent was obtained from the participating parents after they were provided with written information sheets by nursing staff, which were signed just before receiving NADA.

It was essential to clarify that the parents participating in this program were considered healthy companions rather than patients. This distinction was crucial as it alleviated some ethical concerns regarding the treatment of individuals who may be vulnerable due to their infant's medical condition. Furthermore, in compliance with the General Data Protection Regulation (GDPR), the program did not involve the collection of sensitive personal information from participants such as hospital records. This adherence to privacy regulations ensured that parents' rights and confidentiality were upheld throughout the intervention.

NADA therapy was administered solely by two certified NADA practitioners, ensuring that the intervention was delivered according to the established standards and guidelines set forth by the NADA center. This adherence to professional guidelines not only enhanced the quality of care provided but also reinforced the ethical commitment to providing safe and effective treatments.

Participation in the NADA intervention was entirely voluntary, allowing parents of infants in the NICU to make informed decisions about their involvement. This voluntary nature respected the autonomy of the parents and acknowledged their right to choose whether to engage with the intervention. The departmental leadership at the university hospital granted ethical permission to trial NADA, with a limited group of parents to evaluate its relevance and utility as a supportive resource for those with hospitalized infants. This pilot approach was ethically sound, as it sought to gather data on the effectiveness of the intervention while minimizing potential risks to participants.

### 2.1 Data analysis

#### 2.1.1 Quantitative analysis

Changes in scores from pre- to post-assessment were evaluated for the items pertaining to stress, sleep, and physical well-being.

A mixed regression was used with a random intercept by each individual to analyze changes in scores from before to after NADA.

#### 2.1.2 Qualitative analysis

The free-text comments were analyzed thematically to identify themes that could nuance or supplement the questionnaires' answers with quantitative self-reported ratings. Braun and Clarke’s (20) reflexive thematic approach was used as it is flexible and can be used for various qualitative data. All the comments were read and reread by the authors, and the data were then organized in groups by two of the authors (BS, CS). These initial codes were subsequently discussed by the authors in collaboration, where discussions enhanced understanding, reflexivity, and interpretation. In this phase, codes that described similar content were grouped and reviewed in agreement among authors. In the next phase, the authors reviewed and discussed further to identify and define what each theme was about, which resulted in consensus on the findings and final themes. Finally, the analysis was written up, and quotes to illuminate key issues were chosen.

## 3 Results

### 3.1 Quantitative results

In total, 41 parents participated in the study (Table 1). Of these, 23 received fewer than three NADA sessions. Data were collected from 41 parents (33 women and 8 men) who received NADA and completed the post-intervention questionnaire either on the same day or within the next couple of days of their final session. Overall, these participants received an average of 1–16 interventions during their infant’s hospitalization. Among them, 20 had a child born moderate to late preterm.

**Table 1:** Sample characteristics for included participants in the pilot study.

ID	Gender	Knowledge about NADA prior to intervention	Gestation age	Number of NADA sessions	
				<3	>3
1	Female	No	30 + 6		7
2	Female	No	32 + 0		7
3	Female	No	32 + 3		4
4	Female	No	34 + 1		3
5	Female	No	39 + 2	2	
6	Female	No	35 + 4	2	
7	Female	No	34 + 0	1	
8	Male	Yes	35 + 1		3
9	Female	No	33 + 6		6
10	Female	No	32 + 0		7
11	Female	No	35 + 0		3
12	Female	No	34 + 4		3
13	Female	No	34 + 0	1	
14	Female	No	31 + 2		4
15	Female	No	28 + 1		7
16	Female	No	28 + 2		8
17	Female	No	32 + 2		4
18	Female	No	31 + 3		5
19	Male	No	31 + 3	2	
20	Male	No	35 + 4		3
21	Female	Yes	27 + 5		16
22	Female	No	38 + 3		4
23	Female	No	33 + 4	2	
24	Female	No	Missing	2	
25	Female	No	35 + 4	2	
26	Female	No	29 + 1	2	
27	Female	No	41 + 5	2	
28	Female	No	26 + 0	2	
29	Female	No	41 + 1	2	
30	Female	No	35 + 1		3
31	Female	No	35 + 2	2	
32	Female	No	Born at term		4
33	Male	No	27 + 5		9
34	Male	No	Born at term	2	
35	Male	No	34 + 1	2	
36	Male	No	26 + 0	1	
37	Female	No	31 + 6		4
38	Female	No	34 + 0		5
39	Female	No	31 + 2		4
40	Female	No	35 + 4	2	
41	Male	Yes	27 + 5		12

Given the cross-sectional design of the study, causality cannot be established, and the results should be interpreted with caution. However, the results showed changes in levels of stress, sleep quality, and physical well-being among participants before and after receiving the NADA intervention (Table 2). Group means were significantly different, with the *p*-value less than 0.05 (i.e., based on a two-tailed significance level). The level of stress and sleep was lower after intervention with NADA (Table 2). No differences were found related to the actual room in which NADA treatment was conducted.

**Table 2:** Measured changes in stress, sleep, and physical activity before and after the NADA intervention.

Items	Before NADA		After NADA		Difference			
	Mean	SD	Mean	SD	Mean diff	95% CI		*p*-value
Stress score	4.22	0.79	2.27	0.74	−1.951	−2.288	−1.614	0.00
Physical activity	3.34	1.20	1.88	0.64	−1.463	−1.885	−1.042	0.00
Sleep score	4.10	0.83	2.15	0.73	−1.951	−2.294	−1.608	0.00

### 3.2 Qualitative findings

These findings, with short spontaneous evaluative statements on NADA treatment voiced by parents, should be understood in the context of parenting an infant hospitalized in the NICU and the psychological stress commonly experienced by this population.

Parents reported various aspects of their experiences related to receiving NADA intervention through short statements. All parents included free-text comments in the questionnaire. These comments demonstrated that parents associated NADA with positive experiences, physically and mentally, and the overall theme was *an increased feeling of calmness*: “NADA gives me calmness and makes me more relaxed both mentally and physically” (ID 11). Obtaining calmness and relaxation was valued by parents and implies that this is a needed feeling and experienced as a welcomed contribution to the well-being of parents during their NICU stay.

The positive implications of feeling calmness were expressed here: “NADA opens up to it all and gives me serenity to be present in the situation” (ID 9). Being mentally present in the NICU is demanding, and it was implied that emotional withdrawal could serve as a coping mechanism. However, such disengagement could potentially have adverse effects on the relationship with the other parent, the infant, and the collaboration with healthcare professionals. Two subthemes were generated from the comments, each pointing to a distinct aspect of parental NADA experiences: *a psychological booster* and *bodily calmness.* The subthemes elaborated physical and psychological elements that were associated with NADA, all in a positive way. The bodily calmness generated physical effects, and the psychological calmness generated a boosted overview of their life as a family in a NICU context and clarity of thoughts.

### 3.3 A psychological booster

Parents reported that their psychological condition was strengthened in different ways which generated newfound and increased room for reflection and consequently handling the situation mentally. One participant expressed, “NADA helps me to get an overview of my thoughts that otherwise make me anxious and worried” (ID 35)*.* Here, NADA referred to helping to gain an overview in a chaotic mind where the overview was somewhat lost and their thoughts caught up in worry and anxiety. Such a state of calmness and clarity of thought was reported to have helped initiate reflection and to stimulate mental work, exemplified here, “I get to reflect and address some issues that I otherwise would not have been working on if NADA had not helped me to think about it” (ID 12). Being able to reflect and think about the situation seemed to be needed for this participant, and it was remarked as a positive aspect that was considered helpful. Another participant reported:

Coming to NADA, I was so tired of being filled with worry. And really it happened automatically. Instead, I had really good and quite constructive thoughts. So, I have made plans for our admission period here. Now I really think everything is going to be ok (ID4).

The ability and opportunity to reflect and structure thoughts seemed to be a foundation for making choices, where confusion and sadness was a limiting state of mind which was elaborated here: “I was so sad and confused about everything. And at once, when I came back to the room after I got NADA, I totally knew what to do” (ID 17)*.* This statement demonstrated that NADA for this participant was experienced as associated with reduced confusion and sadness, which affected the ability to make choices and act according to the situation, and another noted that after NADA, “the crying stopped and a hopeful feeling of everything is going to be ok” (ID 39) appeared.

Sadness was associated with worries and anxiousness which might have inhibited more energizing feelings comprising hope and positive future expectations. NADA seemed in our data to be experienced as associated with positive regulation of emotions which stimulated mental overview and ability to make choices: “I feel more courageous and happier” (ID 33)*.* Courage could be considered as the opposite of anxiety, which points to an empowering aspect of NADA treatment, to parents who were in an uncertain NICU context where issues of health, sickness, and future were at stake.

### 3.4 Bodily calmness

In addition to the various psychological effects, there were parents who reported on physical effects related to bodily calmness which seemed to entail better abilities to sleep: “NADA gives me a sound and profound sleep, and a feeling of calmness in my body” (ID 37). Sleeping difficulties that had been present even before admission to the NICU could also be helped by NADA treatment during the NICU stay: “This is the first time in six years that I am able to sleep at night” (ID8)*.* Improved sleep was also associated with more mental energy by others and with decreased muscular tensions in the neck.

Some mothers reported that their production of milk increased, which was a bodily reaction that might be related to reduced stress, improved sleep, and psychological calmness. A mother expressed it this way:

I have really relaxed. I snored even though I was awake. I decided to give worries a break, and that just came totally naturally. Instead, I experienced only good constructive thoughts, and I have now planned for the time here at the hospital. It will be okay. After I have slept well, and my milk production has tripled (ID3).

This quote demonstrates how psychological and bodily calmness were experienced as interconnected and constituted inseparable dynamics, which confirmed a holistic non-dualistic view of body and mind.

## 4 Discussion

Given the documented benefits of NADA in other populations experiencing psychological distress (14, 16, 17), we conducted this pilot study with the expectation that the intervention might be transferable to a well-documented high-stress population, parents of preterm or ill infants. The findings from this study suggest that such transferability is possible. Moreover, the results demonstrate that implementing NADA in a NICU setting is feasible. Overall, the intervention was associated with several positive outcomes, and qualitative accounts from parents further elaborated, nuanced, and substantiated these findings.

Following the NADA intervention, participants reported reduced levels of stress, improved sleep, and increased physical well-being. Nevertheless, these findings should be interpreted with caution due to the limitations inherent in the study's design—namely, its pilot nature, cross-sectional methodology, and small sample size—as causal relationships cannot be established. Considering these limitations, outcomes suggest that NADA may enhance mental resources, which can be redirected toward fostering parent–infant attachment and facilitating collaboration with healthcare professionals. These processes are likely to yield both short- and long-term benefits for infants and their parents. Such a dynamic interplay between attachment which serves as a prerequisite for engagement, and mental and bodily well-being is supported by findings from Buchanan et al. (21), which supports the association between auricular acupuncture, reduced anxiety, and enhanced engagement.

However, it is uncertain if the change in, e.g., stress before and after NADA intervention is explained by other reasons than NADA. Such as an improvement in the child's condition, the multifaceted nature of therapeutic encounters that parents can be exposed to while in NICU or resting in a calm setting for 45 min after placement of the needles. An expanding body of literature highlights the therapeutically powerful potential of placebo, particularly in contexts involving psychological and somatic distress (22–24). Placebos have been increasingly recognized as a legitimate biopsychosocial phenomenon, constituting an integral component of the overall therapeutic response even though the effects vary considerably across individuals (22, 24).

Drawing on placebo research, clinical guidelines incorporating three core principles have been developed to facilitate integration of placebo clinical practice settings (24). The first two principles involve the modulation of patient expectations, wherein healthcare professionals convey confidence in the effectiveness of the treatment and provide knowledge for patients. In accordance with these principles, we acknowledge that NADA was communicated to parents in the NICU setting by certified healthcare professionals, thereby conveying a sense of professional credibility and suggesting the potential effectiveness of the intervention. The third principle emphasizes enhancing communication styles to foster a supportive and empathetic relationship between healthcare professionals and patients, which is essential for promoting trust and engagement, which triggers placebo effects. Communicating and collaborating with parents through empathetic and respectful relationships aligns with the core family-centered care values of NICUs in Denmark by developing respectful, informative, and trustful collaboration with parents. Accordingly, this third principle was presumably actively applied in the provision of the NADA, reinforcing a supportive and trust-based therapeutic environment.

The provision of NADA in NICU may be regarded as a holistic approach to supporting parental well-being that comprises a placebo. The findings of our pilot study should therefore be considered within the evidence base of placebo, which posits that observed improvements may not be attributable solely to the specific physiological mechanisms of acupuncture needles.

Research indicates that psychological distress can impact various breastfeeding outcomes, such as delayed secretory activation and reduced duration of exclusive breastfeeding. One suggested physiological mechanism is that psychological distress may hinder the release of oxytocin. Maternal distress can lead to increased serum cortisol levels and reduced insulin sensitivity, both of which are linked to lower milk production. Supporting lactation and breastfeeding goals in women experiencing high levels of psychological distress can greatly benefit both maternal and infant well-being. Investigating stress-reducing programs and policies may improve breastfeeding outcomes (9).

Ziomkiewicz et al. (10) found that perinatal psychosocial stress adversely affected breastmilk composition, specifically by reducing its energy density and altering its fatty acid profile. This points out that in addition to breastmilk volume as essential for the ability to feed the infant, the nutritional quality of the breastmilk is also at stake when mothers are psychosocially stressed. Preterm infants and sick infants are dependent on sufficient nutrition being adequate amounts of high-quality breastmilk to thrive and develop optimally.

## 5 Methodological considerations—limitations

This pilot study was conducted as an observational cross-sectional study. Therefore, it is not plausible to determine any causal relationship between NADA acupuncture and stress, sleep, and well-being. We can read an association. To be able to establish a causal relationship, we need to conduct a randomized controlled trial. However, it is uncertain if the change in, e.g., stress before and after NADA intervention is explained by other reasons than NADA. Such as an improvement in the child's condition, or attention from the parent, from a health professional, the opportunity to rest in a calm room, and a placebo.

For the questionnaire data, the responses might be influenced by social desirability bias (25) with an overrepresentation of positive responses.

Participation in the intervention was voluntary, which may have led to a self-selection bias. It is plausible that more skeptical parents chose not to participate. This potential sampling bias could have influenced the composition of the study population and thereby limited the generalizability of our findings.

As the questionnaire employed in this pilot study was not a validated instrument, there might be limitations in the measures of stress, sleep, and physical well-being. Further studies should ideally use validated questionnaires encompassing themes related to sleep, stress, and physical well-being. Moreover, the recruitment and data collection could include a more equal gender representation in the data.

Having short evaluation statements from parents was a strength to help understand why and how parents benefited (or not) from the intervention. However, further studies could explore more in depth using qualitative interviews. The comments were given after the NADA treatment that they had voluntarily chosen to receive. The questionnaires were administered considering anonymity. To ensure participants felt free to respond without influence, NADA instructors were instructed not to read the completed questionnaires while participants were present.

## 6 Conclusion

This pilot study suggests that NADA may be a promising intervention for reducing stress among parents in the NICU, potentially facilitating the mobilization of mental energy essential for the development of parental attachment and the establishment of parenthood. The findings indicate that NADA could serve as an effective approach to alleviate stress and support parental well-being during this critical and emotionally demanding period. However, this conclusion must be drawn with caution due to the study's limitations. Further research is warranted to substantiate these preliminary findings and to explore the potential effects in greater depth. Future studies should include randomized controlled trials and employ validated measurement tools to assess outcomes such as stress, well-being, sleep quality, and breastfeeding, including breastmilk production.

No adverse effects were reported, and all participants considered NADA as a relevant and valuable intervention. Attention to more equal gender representation should be considered in future studies.

A further potential could be to provide NADA to caregivers in NICUs, such as nurses and doctors.

## Data Availability

The raw data supporting the conclusions of this article will be made available by the authors, without undue reservation.

## Appendix 1 Questionnaire—translated into English.

**Dear parents,**

Please mark on the scale below before and after your NADA course.

1 means no problems, and 5 means very affected.

**Sleep:** To what extent is your sleep affected by your current situation?

**Before NADA:** 1__________2__________3__________4__________5__________

**After NADA:** 1__________2__________3__________4__________5__________

**Stress/Anxiety:** To what extent is your mental well-being affected by your current situation?

**Before NADA:** 1__________2__________3__________4__________5__________

**After NADA:** 1__________2__________3__________4__________5__________

**Physical well-being:** To what extent is your physical condition affected by your current situation (e.g., muscle tension, pain experience, etc.)?

**Before NADA:** 1__________2__________3__________4__________5__________

**After NADA:** 1__________2__________3__________4__________5__________

Total score:_____(to be filled out by the therapist)

**Please describe in your own words how you experience the effect of NADA:**

**—**

**—**

**—**

**Do you think NADA is a relevant offer for hospitalized parents in the neonatal unit?**

By signing, I give consent for this information to be used in data collection for the neonatal unit’s NADA project.

Date: Signature:

Data will be anonymized before presenting the results.

**To be filled out by the NADA therapist:**

Parent’s name:

Child’s name (label):

GA/diagnosis:

Hospitalization period:

Relevant history/conditions for the family:

How did the parent hear about the NADA offer on Neo?

Knowledge/experience with NADA:

Location/setting—how is this experienced in terms of calm, darkness/light, disturbances:

Was the child or other relatives in the same room during the treatment?

Number of treatments (write dates):

Other?

NADA therapist’s name:
